# Caffeates and Caffeamides: Synthetic Methodologies and Their Antioxidant Properties

**DOI:** 10.1155/2019/2592609

**Published:** 2019-11-11

**Authors:** Merly de Armas-Ricard, Enrique Ruiz-Reyes, Oney Ramírez-Rodríguez

**Affiliations:** ^1^Laboratory of Chemistry and Biochemistry, Campus Lillo, University of Aysén, Eusebio Lillo 667, Coyhaique 5951537, Aysén, Chile; ^2^Department of Chemistry, Basic Sciences Institute, Technical University of Manabí (Universidad Técnica de Manabí), Av Urbina y Che Guevara, Portoviejo, Manabí, Ecuador

## Abstract

Polyphenols are secondary metabolites of plants and include a variety of chemical structures, from simple molecules such as phenolic acids to condensed tannins and highly polymerized compounds. Caffeic acid (3,4-dihydroxycinnamic acid) is one of the hydroxycinnamate metabolites more widely distributed in plant tissues. It is present in many food sources, including coffee drinks, blueberries, apples, and cider, and also in several medications of popular use, mainly those based on propolis. Its derivatives are also known to possess anti-inflammatory, antioxidant, antitumor, and antibacterial activities, and can contribute to the prevention of atherosclerosis and other cardiovascular diseases. This review is an overview of the available information about the chemical synthesis and antioxidant activity of caffeic acid derivatives. Considering the relevance of these compounds in human health, many of them have been the focus of reviews, taking as a center their obtaining from the plants. There are few revisions that compile the chemical synthesis methods, in this way, we consider that this review does an important contribution.

## 1. Introduction

Polyphenols are secondary metabolites of plants and include a variety of chemical structures, from simple molecules such as phenolic acids to condensed tannins and highly polymerized compounds. The benefits of polyphenols on human health are often ascribed to their potential ability to act as antioxidants [1, 2]. The phenolic derivatives, such as caffeic acid, catechol, catechin, vanillic acid, eugenol, and thymol, act as natural antimicrobial agents. As components of herbs and spices, that often provide unique flavoring properties, many of these compounds have been used by humans for centuries. These agents protect human health and extend the shelf life of foods [3]. Catechol derivateives with antitumor [4–14], antifungal [15] and antibacterial [16–23] activities, among others [24, 25], have been reported in the literature.

There are two fundamental classes of phenolic acids, hydroxycinnamics (C6–C3) and hydroxybenzoics (C6–C1). Caffeic acid (3,4-dihydroxycinnamic acid) is one of the hydroxycinnamate metabolites more widely distributed in plant tissues. It is present in many food sources, including coffee drinks, blueberries, apples, and cider [26], and also in several medications of popular use, mainly those based on propolis. Its derivatives are also known to possess anti-inflammatory [27, 28], antioxidant [29–31], antitumor [32–39] and antibacterial activities [40–42], and can contribute to the prevention of atherosclerosis and other cardiovascular diseases [30, 43].

Although there are many literature reports that address the different caffeate biological activities, much research remains to be done on this family of polyphenols, and new derivatives with potentially higher activity than natural or synthetic products reported can be obtained. In this review, we will show several synthetic methods and the antioxidant activity of these compounds.

## 2. Chemical Synthesis of Caffeic Acid Derivatives

Polyphenol and its derivatives may be obtained through organic synthesis methodologies from caffeic acid itself or from other chemical precursors.

**Figure 1:**
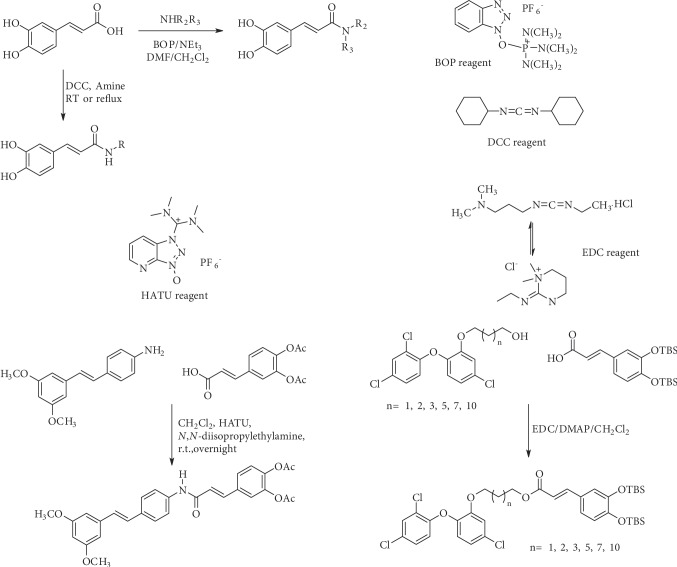
Synthesis of caffeic acid amides using some coupling reagents.

Caffeic acid amides and esters have been synthesized by several methods. One of the most common methods is from caffeic acid using coupling reagents, such as (benzotriazol-1-yloxy)tris(dimethylamino)phosphonium hexafluorophosphate (BOP reagent), dicyclohexylcarbodiimide (DCC), 1-(bis(dimethylamino)methylene)-1*H*-[1,2,3]triazolo[4,5-b]pyridine-1-ium 3-oxide hexafluorophosphate (HATU), and 1-ethyl-3-(3-dimethylaminopropyl)carbodiimide hydrochloride (EDC). Rajan et al. [44], Fu et al. [45], Shi et al. [46], and Jitareanu et al. [47] report the use of BOP to prepare amides from caffeic. Fancelli et al. [48], Arliolo et al. [49], Dai et al. [50], Misra et al. [41], Chen et al. [51], Misra et al. [40], and Liu et al. [52], report the use of DCC. Li et al. [53] report the use HATU, while Kwon et al. [54], Takahashi et al. [55], Chen et al. [56], and Otero et al. [57] report the use of EDC (Figure 1).

**Figure 2:**
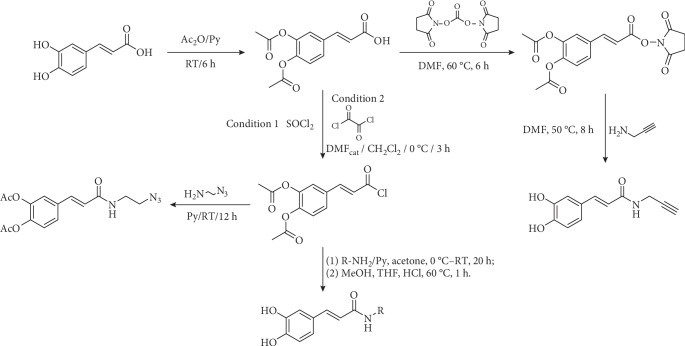
Synthesis of caffeic acid amides using di-*O*-acetyl caffeic acid as intermediate.

**Figure 3:**
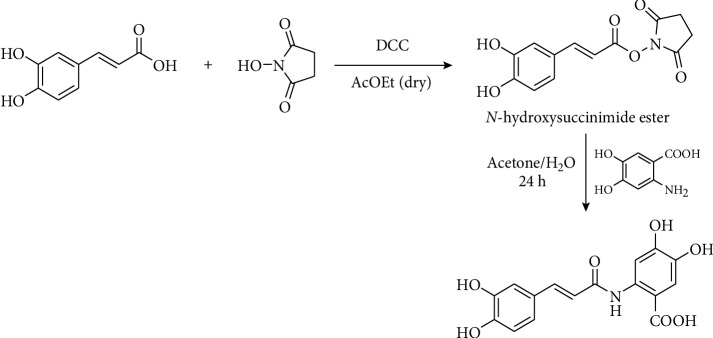
Synthesis of avenanthramides reported by Ishihara et al. [65].

Other methods use acetylated caffeic acid. Caffeic acid is acetylated with acetic anhydride in basic media (pyridine or its derivatives [58–60] or sodium hydroxide [61]) to yield di-*O*-acetyl caffeic acid. This intermediate can be used to prepare amides and esters [62, 63]. Yang et al. [58] synthesize *N*-Propargyl caffeate amide (PACA) transforming this compound into di-*O*-acetyl-caffeic acid *N*-hydroxysuccinimide ester via the reaction with *N,N*′-disuccinimidyl carbonate in DMF. This ester is transformed in propargyl amide by reaction with the corresponding amine, which simultaneously removes the *O*-acetyl groups (Figure 2). *N*-Hydroxysuccinimide esters of *p*-coumaric, ferulic, and caffeic acids are used to transfer hydroxycinnamic moiety to other structures. Stoekigt and Zenk [64] prepared those esters using DCC in dry ethyl acetate and Ishihara et al. [65], using the same protocol, synthesized avenanthramides (Figure 3).

**Figure 4:**
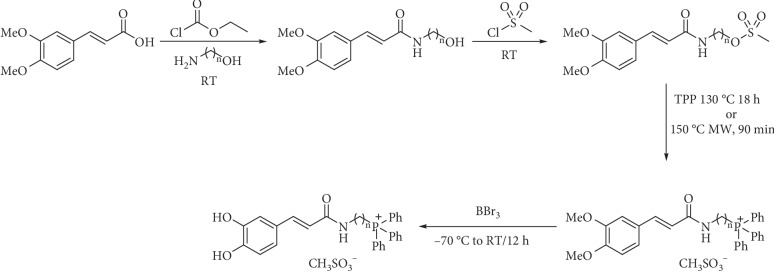
Synthesis of caffeic acid derivatives from methylated acid.

Di-*O*-acetyl caffeic acid can be transformed into amides via acid chloride too [59, 66, 67]. Doiron et al. [67] used acetylated caffeic acid to prepare esters and amides; thionyl chloride with catalytic DMF is preferred to obtain esters, while cold oxalyl chloride in dichloromethane is preferred in the synthesis of the caffeamides (Figure 2). The acetyl protecting groups can be removed under basic [58, 62, 63, 67] or acid [53, 59] mild conditions. Other authors use ethyl chloroformate to obtain amides from protected caffeic acid (Figure 4) [68, 69].

Hydroxyl groups of caffeic acid can be protected by methylation too. Amides can be synthesized by all methods already described. Demethylation reaction is carried out using boron tribromide solution (Figure 4) [69, 70].

Caffeic acid alkyl esters can be obtained by many different pathways. Direct esterification (Fisher method) is one of the most used synthetic strategies to obtain esters with a short alkyl chain, using in the most of case sulfuric acid or *p*-toluenesulfonic acid as catalyst [71–74]. Steverding et al. [75] obtain isoamyl caffeate by this method, refluxing isoamyl alcohol, caffeic acid, and sulfuric acid, for 3 hours. Etzenhouser et al. [73] synthesize different alkyl esters using *p*-toluenesulfonic acid and Dean–Stark trap by the Fisher method. Yang et al. [76] obtained bornyl caffeate by the same method some years later. Sørensen et al. [77] report the esterification of caffeic, ferulic, and coumaric acids, catalyzed by acid either added as the strongly acidic sulfonic resin Amberlite IR-120H or as pure sulfuric acid to the reaction medium. Other authors obtain alkyl esters of ferulic and caffeic acid under microwave irradiation, which is not only faster than using conventional heating methods, but also potentially more efficient, clean, and safe [33]. de Campos et al. [78] synthesized caffeic acid esters by the esterification procedure proposed by Fischer with some modifications, they used acetyl chloride as the source of hydrogen chloride catalyst *in situ*.

There are other methods to synthesize caffeic acid ester. The most common precursors for the synthesis of these compounds are caffeic acid and 3,4-dihydroxybenzaldehyde. From caffeic acid, some authors synthesize alkyl caffeates by nucleophilic displacement of a halogen atom from an alkyl halide in a basic medium [60, 73]. Other authors prepare those compounds using DCC in different conditions [33, 64, 65]. Paracatu et al. [79] report the use of DCC in dioxane and caffeic acid to prepare methyl, butyl, and heptyl caffeate, stirring for 48 h at room temperature with a yield between 53% and 77%. Zhang et al. [80] report the synthesis of many benzyl esters of caffeic acid using DCC in THF, refluxing for 5 hours with much lower yields. Jia et al. [81] use DCC and the esterification reaction was conducted at room temperature for 8 hours. Iqbal et al. [82] obtain guar gum caffeate using DCC in dry DMF at 70°C for 48 hours under inert atmosphere. Other reports show the use of DCC with acetylated caffeic acid. Chyba et al. [83] prepared 4-nitrophenyl caffeate by a combination of standard procedures of organic synthesis and enzymatic deacetylation and used it in assays of caffeoyl esterases.

**Figure 5:**
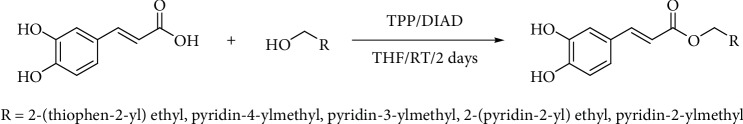
Synthesis of caffeates by Mitsunobu reaction.

Mitsunobu reaction is used in the synthesis of caffeic acid esters too [84–88]. Hajmohamad et al. [87] used this method (triphenylphosphine (TPP) and diisopropyl azodicarboxylate (DIAD) in dry tetrahydrofuran as solvent at room temperature) to obtain several heterocyclic esters of caffeic acid (Figure 5).

**Figure 6:**
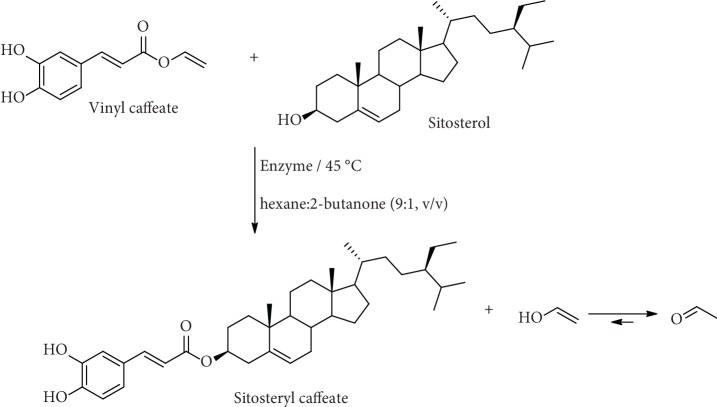
Enzymatic transesterification of vinyl caffeate with sitosterol.

There are many reports of the use of enzymatic methods to obtain esters of caffeic acid. They are mainly transesterification methods. Tan and Shahidi [89] report a novel method for chemoenzymatic synthesis of phytosteryl caffeates through an intermediate vinyl caffeate, which was first chemically produced and subsequently esterified with phytosterols through lipase-assisted alcoholysis (Figure 6). Ten enzymes were initially screened by the authors for their ability in catalyzing the alcoholysis reaction between phytosterols and vinyl caffeate. Lipase from *Candida rugosa* was the only enzyme that successfully catalyzed that alcoholysis reaction.

Pang et al. [90] report the synthesis of propyl caffeate by an enzymatic method. They prepare this compound by transesterification of methyl or ethyl caffeate and 1-propanol using different lipases in an ionic liquid. The best yield was obtained using [Bmim][CF_3_SO_3_] as ionic liquid, Novozym 435 as catalyst, 1 : 20 was the mass ratio methyl caffeate to lipase, and 1 : 5 was the molar ratio methyl caffeate to 1-propanol. The reaction temperature was 60°C.

**Figure 7:**
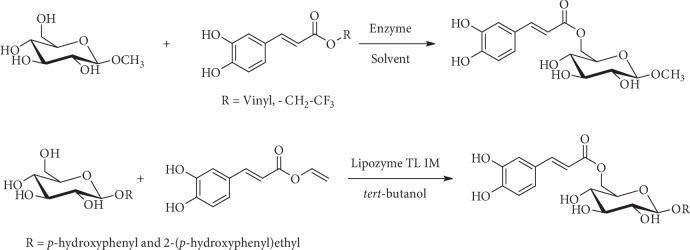
Enzymatic caffeoylation of *β*-*D*-glucopyranosides.

Chyba et al. [91] report the enzymatic caffeoylation of methyl *β*-*D*-glucopyranoside using vinyl and 2,2,2-trifluoroethyl caffeates as caffeoyl donors and a lipase from *Thermomyces lanuginosus* (Lipozyme TL IM). The regioselective formation of methyl 6-*O*-caffeoyl-*β*-*D*-glucopyranoside was obtained using vinyl caffeate in *tert*-butanol and verified with arbutin and salidroside as acceptors (Figure 7).

One of the most common methods reported in the literature for the synthesis of caffeic acid esters uses thionyl chloride as reagent and protected or unprotected caffeic acid in phenolic hydroxyl groups. The most commonly used protection method is acetylation with acetic anhydride. These two methods transform caffeic acid in caffeoyl chloride.

**Figure 8:**
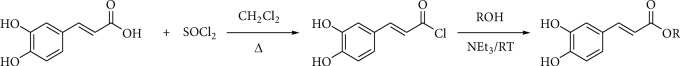
Synthesis of caffeates using thionyl chloride reported by Chou et al. [92].

Reaction with unprotected acid is carried out in a dry solvent, heating to reflux under inert atmosphere (nitrogen or argon). The solvent and SOCl_2_ can be removed under vacuum or not, and then desired alcohol is added under dry conditions. Some authors use a basic medium with alcohol, others do not. The most used solvents are dichloromethane, 1,2-dimethoxyethane (DME) and dioxane (Figure 8) [92–96].

**Figure 9:**
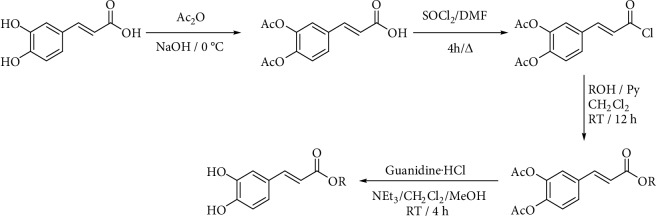
Synthesis of esters using acetylated caffeic acid reported by Sanderson et al. [63].

Many authors prepare acetylated caffeoyl chloride from acetylated caffeic acid by the Vilsmeier–Haack adduct [62, 63, 67, 97], obtained by reaction of thionyl chloride with a catalytic amount of *N*,*N*-dimethylformamide (Figure 9). That carboxylic chloride can be synthesized from the protected acid and thionyl chloride too [98] or using oxalyl chloride and DMF in dichloromethane [61].

Methylated caffeic acid is also used in the synthesis of esters. These esters can be synthesized by all methods already described [99].

**Figure 10:**
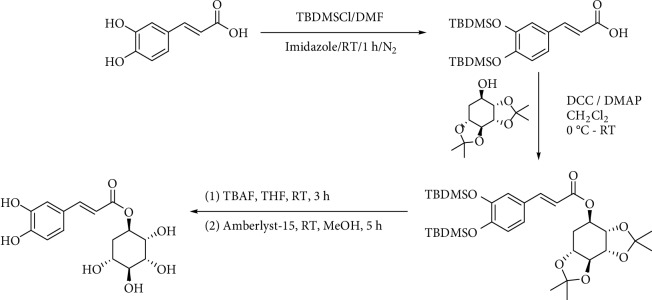
Silylation of hydroxyl groups in caffeic acid reported by Rattanangkool et al. [100].

Silylation is another method to protect phenolic hydroxyl groups in caffeic acid [57, 100, 101]. Rattanangkool et al. [100] use *tert*-butyldimethylsilyl chloride (TBDMSCl) to do that and deprotection was carried out in tetrahydrofuran with TBAF at room temperature for 3 h (Figure 10).

**Figure 11:**
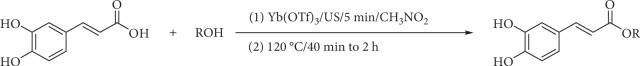
Synthesis of caffeic acid esters using ytterbium triflate as catalyst.

Xie et al. [102] report a convenient and practical catalytic method for the preparation of caffeic acid esters with high efficiency using ytterbium triflate in nitromethane without any other auxiliary reagents. They obtained between 40% and 60% isolated yields without water removal (Figure 11).

Synthesis methods that do not use caffeic acid or its protected derivatives as starting substrates use commonly 3,4-dihydroxybenzaldehyde and by condensation or Wittig reactions obtain the desired compounds.

**Figure 12:**
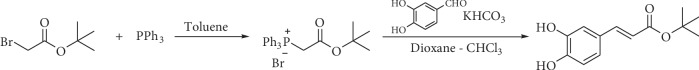
Synthesis of *t*-butyl caffeate by Wittig procedure (Etzenhouser et al. [73]).

Wittig reaction can be used to obtain esters or amides [73, 101, 103]. The most commonly used reagents are esters and amides of *α*-haloacetic acid, which, by reaction with triphenylphosphine, produce the corresponding phosphonium salt (Figure 12).

**Figure 13:**
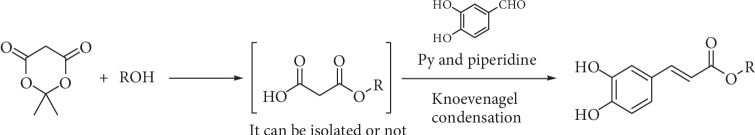
Synthesis of caffeic acid derivatives using meldrum's acid.

One of the most common condensation methods employs a monoester of malonic acid, which can be isolated or not. This method involves two reactions; the first is the synthesis of malonic acid monoester from meldrum's acid and desired alcohol. The second is the Knoevenagel condensation of malonic acid monoester with 3,4-dihydroxybenzaldehyde in the presence of a base (pyridine and piperidine in most cases) at room temperature for 12–24 h (Figure 13) [68, 74, 94, 103–110].

**Figure 14:**
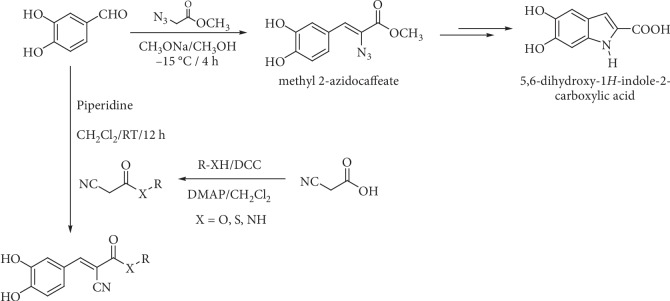
Synthesis of caffeic acid derivatives substituted in vinylic position.

Knoevenagel condensation of 3,4-dihydroxybenzaldehyde with other compounds can be performed to obtain other derivatives of caffeic acid, some of them substituted in vinylic carbons. Sechi et al. [111, 112] synthesized 2-azido-3-(3,4-dihydroxy-phenyl)-acrylic acid methyl ester (methyl 2-azidocaffeate) as an intermediary to obtain 5,6-dihydroxy-1*H*-indole-2-carboxylic acid (Figure 14). Rodrigues et al. [113] synthesized cyanoacetic acid derivatives (esters, amides, and thioesters) and obtained the caffeic acid derivatives by Knoevenagel condensation of these compounds with 3,4-dihydroxybenzaldehyde under basic conditions (Figure 14).

## 3. Antioxidant Activity

The reactive oxygen species (ROS) such as superoxide anion radical, hydrogen peroxide, and hydroxyl radical are generated in all cells due to both endogenous metabolic processes as exogenous stimuli. However, cells are usually able to reduce the oxidative potential of ROS by activating several antioxidant systems. In plants, one of these defense systems are polyphenols, making this family of compounds a target for the search for applications in the food and pharmaceutical industries. In this context, it has been reported that some compounds such as caffeic acid (entry 1, Table 1) and its derivatives have antioxidant properties [114]. According to Chung et al. [115] caffeic acid has an antioxidant effect against the oxidative lesions that are produced in the gill cells of trout. A concentration-dependent inhibition of iron-catalyzed lipid peroxidation is, moreover, exerted by esters, as octyl caffeate (0.1–1.0 mM), in rat brain homogenates. It has been shown to have a potent antioxidant when the nitric oxide synthase (iNOS) expression is induced by means of lipopolysaccharides (LPS), and interferon-*γ* (IFN-*γ*) in cultured primary rat aortic smooth muscle cells (RASMC) *in vitro*, in addition to induced hypotension by means of LPS *in vivo* [116]. Recently, Kyselka et al. [117] have reported that caffeic acid and methyl caffeate (entry 2, Table 1) showed the highest reduction rate against the oxidation reaction with the 1,1-diphenyl-2-picrylhydrazyl radical (DPPH^•^) showing better results as an antioxidant than other phenolic compounds.

Chapado et al. [86] reported the synthesis of dihydroxyphenetyl caffeate (entry 10, Table 1), among others esters structurally related to rosmarinic acid (entry 11, Table 1), and evaluated their antioxidant activity against DPPH^•^. Those compounds showed better antiradical activity than their precursors (dihydroxyphenetyl alcohol, caffeic, protocatechuic, and gallic acids) and rosmarinic acid. Taguchi et al. [118] also reported the ability of rosmarinic acid derivates as antioxidants (DPPH radical scavenging assay), along with that of certain esters (entries 1–9, Table 1) and amides (entries 15 and 16, Table 1) of caffeic acid. These authors found 61%–63% of DPPH radical scavenging activity for alkyl esters and 2-(3,4-dihydroxyphenyl)ethyl caffeamide, while *ω*-OH esters and pentyl amide showed 53%–55% of activity. The results suggested that those compounds without catechol moiety (entry 17, Table 1) showed low inhibition percentages even at very high concentrations (<10% at 500 *µ*M concentration). Therefore, the presence of catechol ring is important in the scavenging action of ROS species. However, they could not find a specific structural feature of caffeic acid-type compounds, having an account that caffeic acid itself has significant antioxidant activity. Amoussa et al. [119] report the antioxidant activity of 3-caffeoylbetulinic acid (entry 12, Table 1), it showed significant antioxidant activity with an IC_50_ of 3.57  *μ*g/mL compared to quercetin (control) 1.04  *μ*g/mL.

Esters obtained from phenolic hydroxyl groups of caffeic acid also show antioxidant activity. Gandolfi et al. [120] report the radical scavenging activity (RSA) towards DPPH of 3-[(2*E*,4*E*,6*E*)-octa-2,4,6-trienoyl]caffeic acid and 4-[(2*E*,4*E*,6*E*)-octa-2,4,6-trienoyl]caffeic acid (entry 13, Table 1) and 3,4-di-[(2*E*,4*E*,6*E*)-octa-2,4,6-trienoyl]caffeic acid (entry 14, Table 1). They show, in general, esterification with caffeic acid led to a higher increase in RSA, although the diester did not show higher activity than caffeic acid.

Rajan et al. [44] synthesized caffeic acid amides and studied their antioxidant properties as lipid peroxidation inhibitory activity. Caffeic acid anilides were very efficient antioxidants with IC_50_ of 0.3 *µ*M (entries 1 and 2, Table 2). The aliphatic amides also showed activity, and were slightly lower than the anilides (entries 3 and 4, Table 2). These amides showed antioxidant activity comparable with standard antioxidants such as Trolox, caffeic acid, and quercetin (entries 5–7, Table 2). *p*-coumaric acid amides are 10 times less active, which suggest that the catechol ring has influence on the antioxidant activity [44].

Lira et al. [121] studied *in vitro* oxidant and antioxidant activity of isopropyl caffeate in the presence of phenylhydrazine and Reactive Oxygen Species. They showed that no hemoglobin oxidation was observed at concentrations lower than 100 *µ*g/mL (compared to the negative control), but it could not prevent the oxidation of hemoglobin in the presence of phenyl hydrazine. Therefore, there is not significant oxidant power in this substance. Furthermore, the authors noted that isopropyl caffeate was able to react with ROS at concentrations of 10, 50, 100, and 250 *μ*g/mL. They also discovered that the hemolysis induced by hydrogen peroxide was reduced when compared to the positive control group (Hb + H_2_O_2_), and finally, isopropyl caffeate shows a greater antioxidant power than vitamin C.

On the other hand, Pérez-Cruz et al. [122] have reported the antioxidant activity of coumarin derivatives with phenolic acid moieties against the biologically relevant ROS using assays as oxygen radical absorbance capacity fluorescein (ORAC-FL), the ferric reducing ability of plasma (FRAP), electronic spin resonance (ESR), and cellular antioxidant activity (CAA). These compounds showed better ORAC-FL values than Trolox, and two or three times more than coumarin moiety alone. Therefore, the polyphenol inclusion in the coumarin scaffold contributes to the antioxidant capacity. The evaluation of ^•^OH scavenging was done by ESR, and the radical-scavenging values indicated that the coumarin caffeic and gallic derivatives were similar and better than the coumarin moiety, and attained values of approximately 99%. The FRAP assay showed that derivatives have values between two and three times higher than their coumarinic precursor, suggesting that the inclusion of phenolic moieties in the original coumarinic scaffold increases the reducing capacity.

Doiron et al. [123] synthesized some propargyl and allyl esters of caffeic acid and assayed their antioxidant activity by employing 2,2-diphenyl-1-picrylhydrazyl (DPPH). All of them showed a good ability radical scavenging with most having IC_50_ values in the range of 10–15 *μ*M, being similar to the radical scavenging activity of caffeic acid (15.3 *μ*M, entry 1 Table 1) and CAPE (11.9 *μ*M, entry 18 Table 1). The authors found little differences in radical scavenging activity of monovalent esters of both series (propargyl and allyl esters; entries 3, 19–26, and 28–34, Table 1; respectively). These findings indicate that the antioxidant activity of caffeic acid catecholic ring is practically insensitive to changes that do not directly alter it. Derivatives with two catechol rings (dimers of caffeic acid derivatives, entries 27 and 35, Table 1) have twice as much antioxidant activity as monoesters.

Finally, Misra et al. [41] report on the antioxidant and antibacterial activities of a new caffeamide series (entries 36–38, Table 1). They observed that amides having electron withdrawing group attached had lower EC_50_ value than caffeic acid. They behave more potent antioxidant with respect to caffeic acid, whereas electron donating moiety attached with caffeamide had a higher EC_50_ value compared to caffeic acid, as expected.

## 4. Conclusions

In addition to extraction from natural sources, there are cheap and easy to make synthetic methods for obtaining caffeic acid derivatives. These methods, unlike the extractive ones, could provide enough quantity of caffeic acid derivatives for their multiple uses, besides guaranteeing the preservation of the plants as a natural resource. In this review, the alternatives for the synthetic obtaining of esters and amides of caffeic acid by simple synthetic methods are shown.

## Figures and Tables

**Table 1:** Antioxidant activity of caffeic acid derivatives against DPPH^•^.

Entry	Compound	Structure		DPPH radical scavenging activity^a^	Reference
1	Caffeic acid	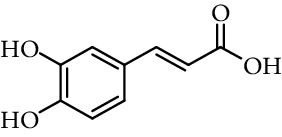		63% (500 *μ*M)	[118]
				0.17 *μ*mol/*μ*mol DPPH	[117]
				0.17 mol/mol DPPH	[86]
				IC_50_ 15.3 *μ*M	[123]
				EC_50_ 30.88 *µ*M	[41]
2	Caffeic acid methyl ester	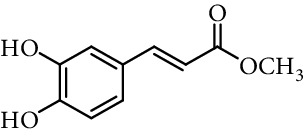		62% (500 *μ*M)	[118]
				0.17 *μ*mol/*μ*mol DPPH	[117]
3	Caffeic acid allyl ester	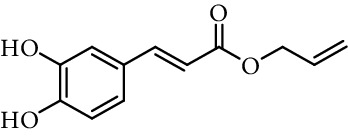		64% (500 *μ*M)	[118]
				IC_50_ 12.3 *µ*M	[123]
4	Caffeic acid propyl ester	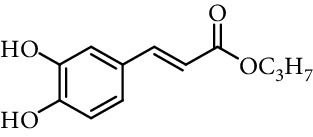		63% (500 *μ*M)	[118]
5	Caffeic acid butyl ester	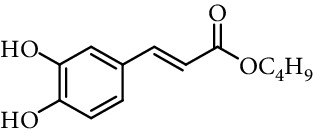		62% (500 *μ*M)	[118]
6	Caffeic acid pentyl ester	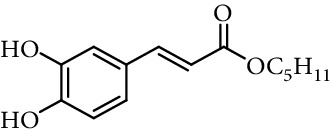		62% (500 *μ*M)	[118]
7	Caffeic acid hexyl ester	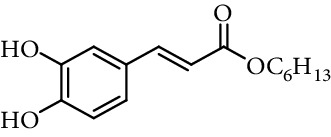		61% (500 *μ*M)	[118]
8	Caffeic acid heptyl ester	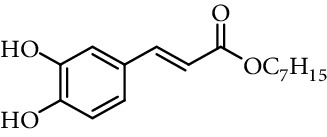		63% (500 *μ*M)	[118]
9	Caffeic acid nonyl ester	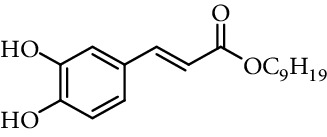		61% (500 *μ*M)	[118]
10	Caffeic acid 3,4-dihydroxyphenetyl ester	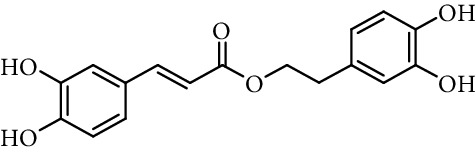		0.09 mol/mol DPPH	[86]
11	Rosmarinic acid	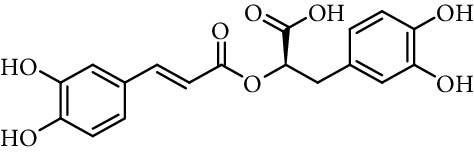		0.12 mol/mol DPPH	[86]
				61% (500 *μ*M)	[118]
12	3-Caffeoylbetulinic acid	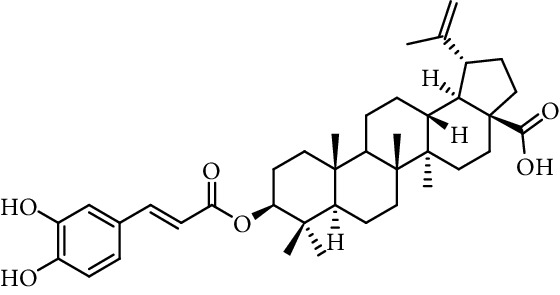		IC_50_ of 3.57 *μ*g/mL	[119]
13	3- and 4-[(2*E*,4*E*,6*E*)-octa-2,4,6-trienoyl]caffeic acid	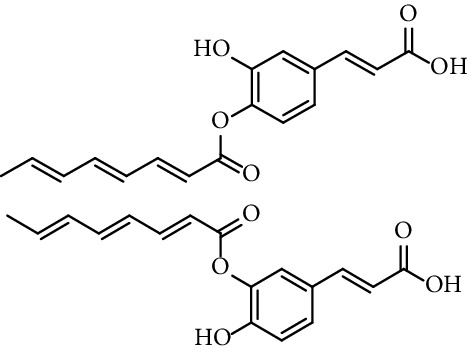		96.25% (5 mM)	[120]
14	3,4-Dioctatrienoyl caffeic acid	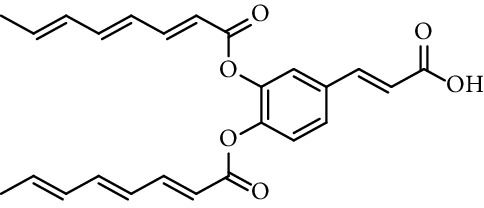		100% (5 mM)	[120]
15	*N*-(3,4-Dihydroxyphenethyl) caffeamide	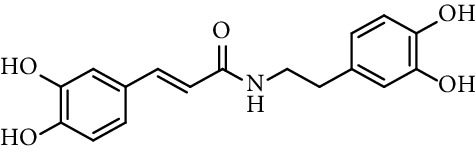		64% (500 *µ*M)	[118]
16	*N*-Pentyl caffeamide	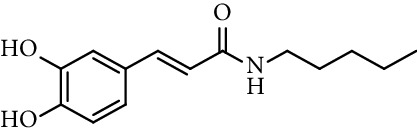		53% (500 *µ*M)	[118]
17	2-Phenylethyl cinnamate	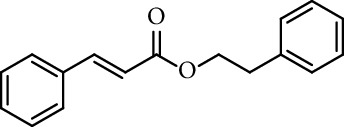		<10%	[118]
18	Caffeic acid phenetyl ester (CAPE)	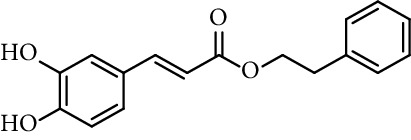		IC_50_ 11.9 *µ*M	[123]
19	Caffeic acid propargylic esters	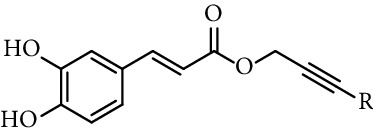	R = H	IC_50_ 11.1 *µ*M	[123]
20			R = Ph	IC_50_ 12.7 *µ*M	[123]
21			R = 4-CH_3_-Ph	IC_50_ 11.5 *µ*M	[123]
22			R = 4-CH_3_O-Ph	IC_50_ 13.7 *µ*M	[123]
23			R = 4-NO_2_-Ph	IC_50_ 10.6 *µ*M	[123]
24			R = 4-F-Ph	IC_50_ 10.7 *µ*M	[123]
25			R = 1-naphthyl	IC_50_ 13.7 *µ*M	[123]
26			R = 4-Ph-Ph	IC_50_ 15.0 *µ*M	[123]
27	Bis-caffeoyl propargyl derivative	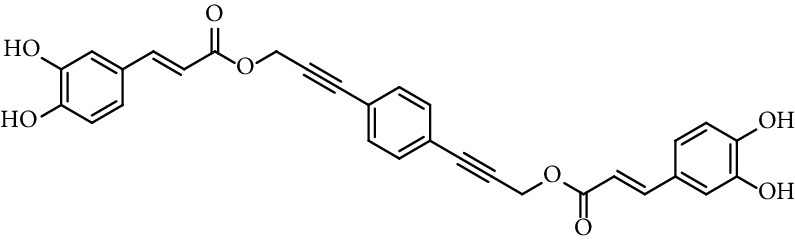		IC_50_ 5.6 *µ*M	[123]
28	Caffeic acid allyl esters	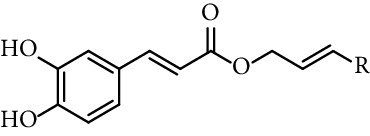	R = Ph	IC_50_ 12.4 *µ*M	[123]
29			R = 4-CH_3_-Ph	IC_50_ 13.1 *µ*M	[123]
30			R = 4-CH_3_O-Ph	IC_50_ 11.6 *µ*M	[123]
31			R = 4-NO_2_-Ph	IC_50_ 12.3 *µ*M	[123]
32			R = 4-F-Ph	IC_50_ 12.31 *µ*M	[123]
33			R = 1-naphthyl	IC_50_ 13.0 *µ*M	[123]
34			R = 4-Ph-Ph	IC_50_ 12.03 *µ*M	[123]
35	Bis-caffeoyl allyl derivative	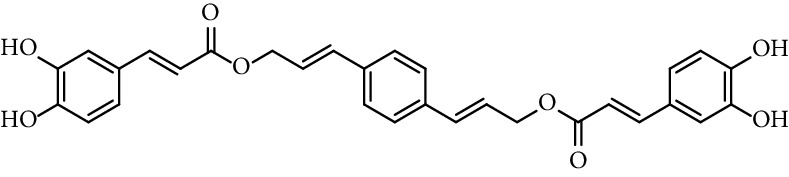		IC_50_ 6.1 *µ*M	[123]
36	*N*-(3,5-Dichloro-4-hydroxyphenyl)-caffeamide	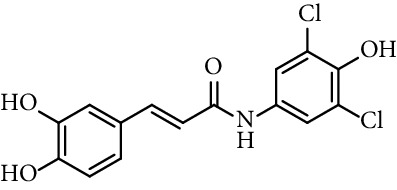		EC_50_ 5.51 *µ*M	[41]
37	*N*-(4-Nitrophenyl)-caffeamide	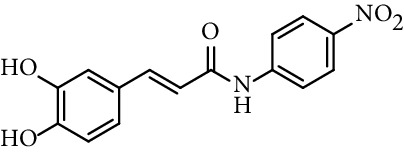		EC_50_ 7.21 *µ*M	[41]
38	*N*-(4-Aminophenyl)-caffeamide	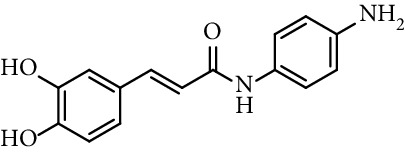		EC_50_ 36.01 *µ*M	[41]

^a^IC_50_: Inhibitory concentration, EC_50_: Effective concentration.

**Table 2:** Lipid peroxidation inhibitory activity of caffeic acid amides and related compounds.

Entry	Compound	Structure	IC_50 _(*µ*M)^a^	Reference
1	*N*-(2-Hydroxyphenyl) caffeamide	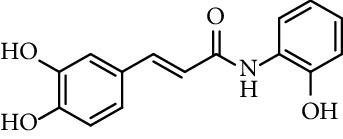	0.29	[44]
2	*N*-Phenyl caffeamide	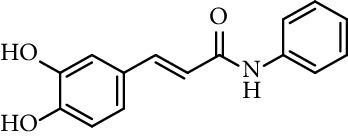	0.38	[44]
3	*N*-(3,4-Dihydroxyphenethyl) caffeamide	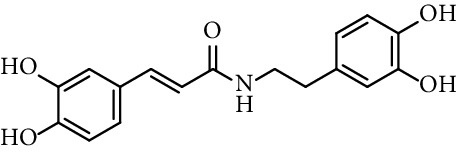	0.59	[44]
4	*N*-Isopentyl caffeamide	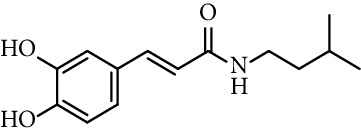	1.4	[44]
5	Trolox	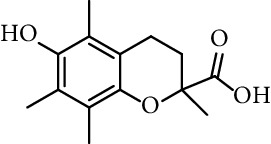	2.8	[44]
6	Caffeic acid	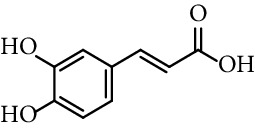	3.3	[44]
7	Quercetin	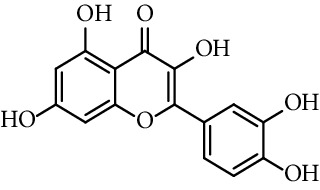	0.95	[44]

^a^The antioxidant activity of each compound was expressed as IC_50_ value, i.e., the concentration in *µ*M necessary to inhibit TBARS formation by 50%, and was calculated from the corresponding log-dose inhibition curve.
